# Diffusion-Weighted Imaging in 3.0 Tesla Breast MRI: Diagnostic Performance and Tumor Characterization Using Small Subregions vs. Whole Tumor Regions of Interest

**DOI:** 10.1371/journal.pone.0138702

**Published:** 2015-10-12

**Authors:** Otso Arponent, Mazen Sudah, Amro Masarwah, Mikko Taina, Suvi Rautiainen, Mervi Könönen, Reijo Sironen, Veli-Matti Kosma, Anna Sutela, Juhana Hakumäki, Ritva Vanninen

**Affiliations:** 1 Kuopio University Hospital, Diagnostic Imaging Centre, Department of Clinical Radiology, Kuopio University Hospital, Kuopio, Finland; 2 Kuopio University Hospital, Department of Pathology, Kuopio University Hospital, Kuopio, Finland; 3 University of Eastern Finland, Institute of Clinical Medicine, School of Medicine, Department of Clinical Radiology, Kuopio University Hospital, Kuopio, Finland; 4 University of Eastern Finland, Institute of Clinical Medicine, School of Medicine, Department of Clinical Pathology and Forensic Medicine, Kuopio University Hospital, Kuopio, Finland; 5 University of Eastern Finland, Cancer Center of Eastern Finland, Kuopio, Finland; Shenzhen institutes of advanced technology, CHINA

## Abstract

**Introduction:**

Apparent diffusion coefficient (ADC) values are increasingly reported in breast MRI. As there is no standardized method for ADC measurements, we evaluated the effect of the size of region of interest (ROI) to diagnostic utility and correlation to prognostic markers of breast cancer.

**Methods:**

This prospective study was approved by the Institutional Ethics Board; the need for written informed consent for the retrospective analyses of the breast MRIs was waived by the Chair of the Hospital District. We compared diagnostic accuracy of ADC measurements from whole-lesion ROIs (WL-ROIs) to small subregions (S-ROIs) showing the most restricted diffusion and evaluated correlations with prognostic factors in 112 consecutive patients (mean age 56.2±11.6 years, 137 lesions) who underwent 3.0-T breast MRI.

**Results:**

Intra- and interobserver reproducibility were substantial (κ = 0.616–0.784; Intra-Class Correlation 0.589–0.831). In receiver operating characteristics analysis, differentiation between malignant and benign lesions was excellent (area under curve 0.957–0.962, cut-off ADC values for WL-ROIs: 0.87×10^−3^ mm^2^s^-1^; S-ROIs: 0.69×10^−3^ mm^2^s^-1^, P<0.001). WL-ROIs/S-ROIs achieved sensitivities of 95.7%/91.3%, specificities of 89.5%/94.7%, and overall accuracies of 89.8%/94.2%. In S-ROIs, lower ADC values correlated with presence of axillary metastases (P = 0.03), high histological grade (P = 0.006), and worsened Nottingham Prognostic Index Score (P<0.05). In both ROIs, ADC values correlated with progesterone receptors and advanced stage (P<0.01), but not with HER2, estrogen receptors, or Ki-67.

**Conclusions:**

ADC values assist in breast tumor characterization. Small ROIs were more accurate than whole-lesion ROIs and more frequently associated with prognostic factors. Cut-off values differed significantly depending on measurement procedure, which should be recognized when comparing results from the literature. Instead of using a whole lesion covering ROI, a small ROI could be advocated in diffusion-weighted imaging.

## Introduction

Breast cancer is the most common cancer affecting women and is associated with high mortality rates [1, 2]. The role of dynamic contrast-enhanced magnetic resonance imaging (DCE-MRI) in local staging and breast lesion characterization is well established [3]. Although the high sensitivity of DCE-MRI ranging from 85% to 100%, lower specificities (37% to 88%) have been achieved [4, 5]. Diffusion-weighted imaging (DWI) and the evaluation of apparent diffusion coefficient (ADC) values improves differential value of MRI, amends the positive predictive value, and reduces unnecessary biopsies [6].

Thus far, correlations between ADC values and traditional prognostic factors of breast cancer have been reported infrequently, especially using 3.0-T MRI [7–15]. Compared with 1.5-T MRI, 3.0-T imaging allows higher signal-to-noise ratios, improved spatial resolution, and faster scanning, resulting in improved anatomical detail [16] and greater diagnostic accuracy in breast lesion diagnostics [17], and therefore may be able to predict malignancy of lesions more accurately. As a quantitative parameter, ADC values may also be used for radiogenomic explorations. Imaging could provide with information about tumor heterogeneity non-invasively [18] and predict clinical and molecular characteristics in radiogenomic approaches [19, 20].

However, the procedure for ADC measurements in breast lesions has not been standardized. As a result, a range of different sizes and methods are used to place the region of interest (ROI) [9, 21–23]. Size and positioning of ROIs affect both ADC levels and the reproducibility of measurements [24]. When multiple small ROIs are employed in the subregions that display the most restricted diffusion instead of the entire tumor, the measured ADC values more accurately represent the most aggressive tissue component used in histopathological diagnosis [11, 25, 26]. Use of a small ROI would also parallel the DCE kinetic curve analysis, which according to the Breast Imaging Reporting and Data System (BI-RADS^®^) is instructed to be trained on the most suspicious region of enhancement within a lesion [27].

The aims of the present study were a) to assess the diagnostic accuracy of 3.0-T DWI in the differentiation of benign and malignant lesions, b) to compare the diagnostic performance of ADC measurements from a ROI covering the whole tumor (WL-ROI) vs. a small ROI (S-ROI) placed in the most aggressive appearing subregion, and c) to evaluate the association of the 3.0-T ADC values with traditional prognostic factors.

## Materials and Methods

### Patients and Study Design

The Institutional Ethics Board of Kuopio University Hospital approved this prospective study; the Chair of the Hospital District waived the need for written informed consent for the retrospective analyses of the breast MRIs. No pathological, clinical or radiological data were available at the time of patient selection. All data was measured and analysed blinded to pathological and clinical records. Consecutive patients admitted to Kuopio University Hospital between April 2011 and April 2014 who were referred for 3.0-T breast MRI either with clinical indications by the European Society of Breast Cancer Specialists working group (EUSOMA) [28] or with consideration of oncoplastic surgery according to national guidelines were included. Patients with suspicious findings in MRI or conventional triple testing (mammography, ultrasound, and clinical examination) were further biopsied. Inclusion criteria for the present study were 1) a minimum lesion size of 0.5 cm on DCE; 2) verification of all evaluated lesions using core-needle biopsies or surgically harvested samples; and 3) lesion detectable on DWI. Before ADC values were measured, three MRI examinations were excluded owing to motion artifacts. The study population consisted of 112 women (mean age 56.19±11.56 years, range 28–82 years) with 152 biopsy-proven lesions, of which 137 were DWI-visible. Patient and lesion characteristics are presented in Tables 1 and 2.

**Table 1:** Patient demographics and tumor characteristics.

Characteristic	N (%)
Patients	112
Total number of lesions	137
Age (years)	56.19±11.56
Tumor classification	
Malignant, invasive	104 (75.9)
In situ	10 (7.3)
Benign	23 (16.8)
Lesion focus	
Mass	111 (81.0)
Non-mass	26 (19.0)
Stage^1^	
S0	10 (10.3)
S1	34 (35.1)
S2	30 (30.9)
S3	22 (22.7)
S4	1 (1.0)
Axillary lymph node metastasis^2^	
Positive	36 (38.7)
Negative	57 (61.3)
T Classification^1^	
Tis	10 (9.0)
T1	51 (45.9)
T2	41 (36.9)
T3	7 (6.3)
T4	2 (1.8)
Tumor grade^2^	
G1	19 (19.4)
G2	48 (49.0)
G3	31 (31.6)
Estrogen receptor^2^	
Positive	86 (85.1)
Negative	15 (14.9)
Progesterone receptor^2^	
Positive	80 (79.2)
Negative	21 (20.8)
Triple negative^2^	11 (10.9)
HER2^2^	
Positive	18 (17.3)
Negative	86 (82.7)
Ki-67 expression^2^	
Low	26 (25.2)
Moderate	28 (27.2)
High	49 (47.6)
Malignant lesion histology	
Ductal	84 (61.3)
Lobular	12 (8.8)
Tubulolobular	7 (5.1)
Tubular	1 (0.7)
Premalign lesion histology	
Ductal carcinoma in situ	10 (7.3)
Benign lesion histology	
Fibroadenoma	8 (5.8)
Fibrocystic changes	7 (5.1)
Papilloma	4 (2.9)
Imflammatory changes	4 (2.9)

^1^ all malignant lesions

^2^ invasive carcinomas.

**Table 2:** Association of apparent diffusion coefficient (ADC) values (× 10^−3^ mm^2^s^-1^) measured by Observer 1 with pathologic and prognostic factors in breast lesions, using the whole lesion and small regions of interest (ROIs).

		Whole lesion ROI		Small ROI	
	N	ADC mean±SD	P	ADC mean±SD	P
Lesion classification			0.000		0.000
Malignant	114	0.61±0.20		0.44±10.17	
Benign, not premalignant	23	1.10± 0.22		0.93±0.26	
Malignant lesion invasiveness			0.001		0.001
Invasive	104	0.59±0.18		0.41±0.14	
Premalignant	10	0.86±0.24		0.68±0.27	
Non-invasive lesion characterization			0.013		0.022
In situ	10	0.86±0.24		0.68±0.27	
Benign	23	1.10± 0.22		0.93±0.26	
Malignant lesion aggressiveness			ns		0.006
High	85	0.59±0.20		0.42±0.17	
Low	19	0.66±0.18		0.49±0.12	
Estrogen receptor			ns		ns
Positive	86	0.58±0.18		0.41±0.13	
Negative	15	0.63±0.17		0.43±0.14	
Progesterone receptor			0.008		0.046
Positive	80	0.57±0.18		0.40±0.14	
Negative	21	0.66±0.18		0.47±0.13	
HER2 expression			ns		ns
Positive	18	0.62±0.16		0.42±0.11	
Negative	86	0.58±0.19		0.42±0.14	
Triple negative			ns		ns
Yes	11	0.71±0.17		0.71±0.17	
No	90	0.55±0.17		0.55±0.17	
Ki-67 expression			ns		ns
Low	26	0.62±0.20		0.45±0.15	
Moderate	28	0.56±0.17		0.40±0.12	
High	49	0.59±0.19		0.42±0.16	
Axillary lymph node metastasis			ns		0.03
Positive	36	0.55±0.16		0.38±0.12	
Negative	57	0.61±0.19		0.44±0.14	
Lymphovascular invasion			ns		0.043
Positive	35	0.57±0.18		0.39±0.14	
Negative	75	0.60±0.18		0.43±0.14	
Stage			0.008		0.010
0	10	0.86±0.24		0.68±0.27	
1	34	0.67±0.23		0.49±0.20	
2	30	0.60±0.19		0.42±0.15	
3 and 4	23	0.56±0.15		0.39±0.12	
Nottingham Prognostic Index			ns		ns
93% Survival	11	0.62±0.11		0.47±0.10	
85% Survival	22	0.57±0.22		0.42±0.16	
70% Survival	20	0.60±0.17		0.42±0.13	
50% Survival	42	0.58±0.19		0.39±0.14	

ns = not significant.

### Breast MRI Protocol

MRI examinations were performed in the prone position with a 7-element phased-array coil dedicated to breast imaging (Philips Achieva 3.0-T TX, Philips N.V., Eindhoven, The Netherlands). The structural breast MRI protocol consisted of five sequences: 1) T1-weighted fast field echo (TR = shortest; TE (in phase) = 2.3 ms; in-plane resolution 0.48 mm x 0.48 mm; 257 slices; slice thickness 0.7 mm; scanning time 6 minutes (min) 11 s); 2) T2-weighted turbo spin echo (TR = 5000 ms; TE = 120 ms, flip angle 90°; in-plane resolution 0.6 mm x 0.6 mm; 85 slices; slice thickness 2 mm; scanning time 3 min 20 s); 3) short T1-inversion recovery/turbo spin echo (TR = 5000 ms; TE = 60 ms; TI 230 ms; in-plane resolution 1 mm x 1 mm; 90 slices; slice thickness 2 mm; scanning time 5 min 40 s); 4) a dynamic eTHRIVE sequence (TR = shortest; TE = shortest; spectrally adiabatic inversion recovery (SPAIR) fat suppression; dynamic scan time 58.5 s; in-plane resolution 0.96 mm x 0.96 mm; 180 slices; slice thickness 1 mm; with precontrast and six phases after the gadoterate meglumine (0.2 ml/kg, 3 ml/s) injection followed by a saline chaser; and 5) DWI echo planar imaging (TR = shortest; TE = 95 ms; flip angle 90°; SPAIR fat suppression; in-plane resolution 1.15 mm x 1.15 mm; 30 slices; slice thickness 4 mm; diffusion gradients in three directions; scanning time 4 min 8 s) with five respective b factors (0, 200, 400, 600, and 800 s/mm2). The ADC maps were automatically calculated linearly by the method provided by the MRI vendor. Breast radiologists (with 14–20 years of experience in breast radiology) evaluated MRI findings together with mammograms and ultrasound examinations according to the BI-RADS^**®**^ lexicon [27].

### DW Image Analysis

**Fig 1:**
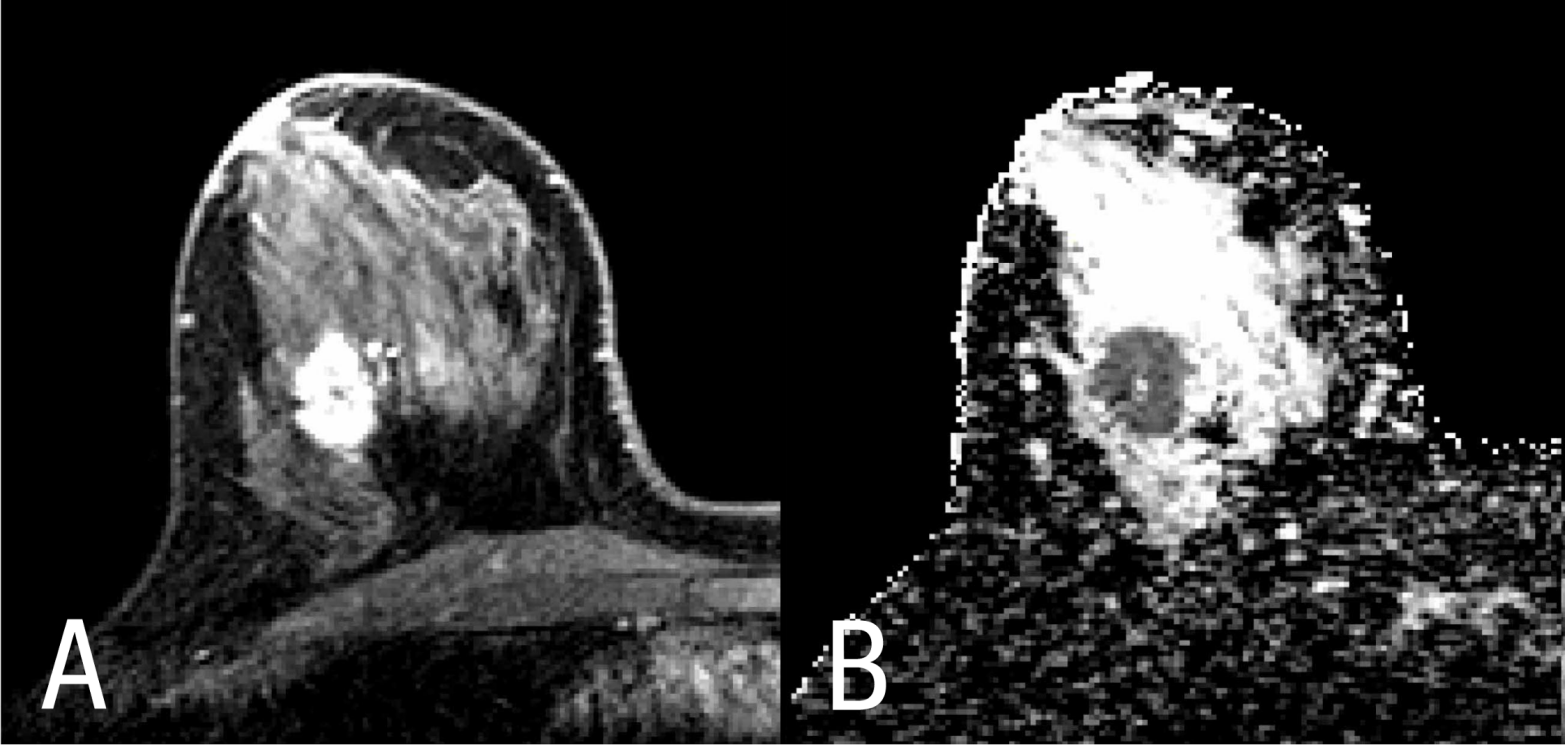
Lesion differentiation in (A) dynamic contrast-enhanced magnetic resonance imaging (MRI, left) and in (B) diffusion-weighted imaging (DWI, right) of the right breast of a 41-year-old woman.

**Fig 2:**
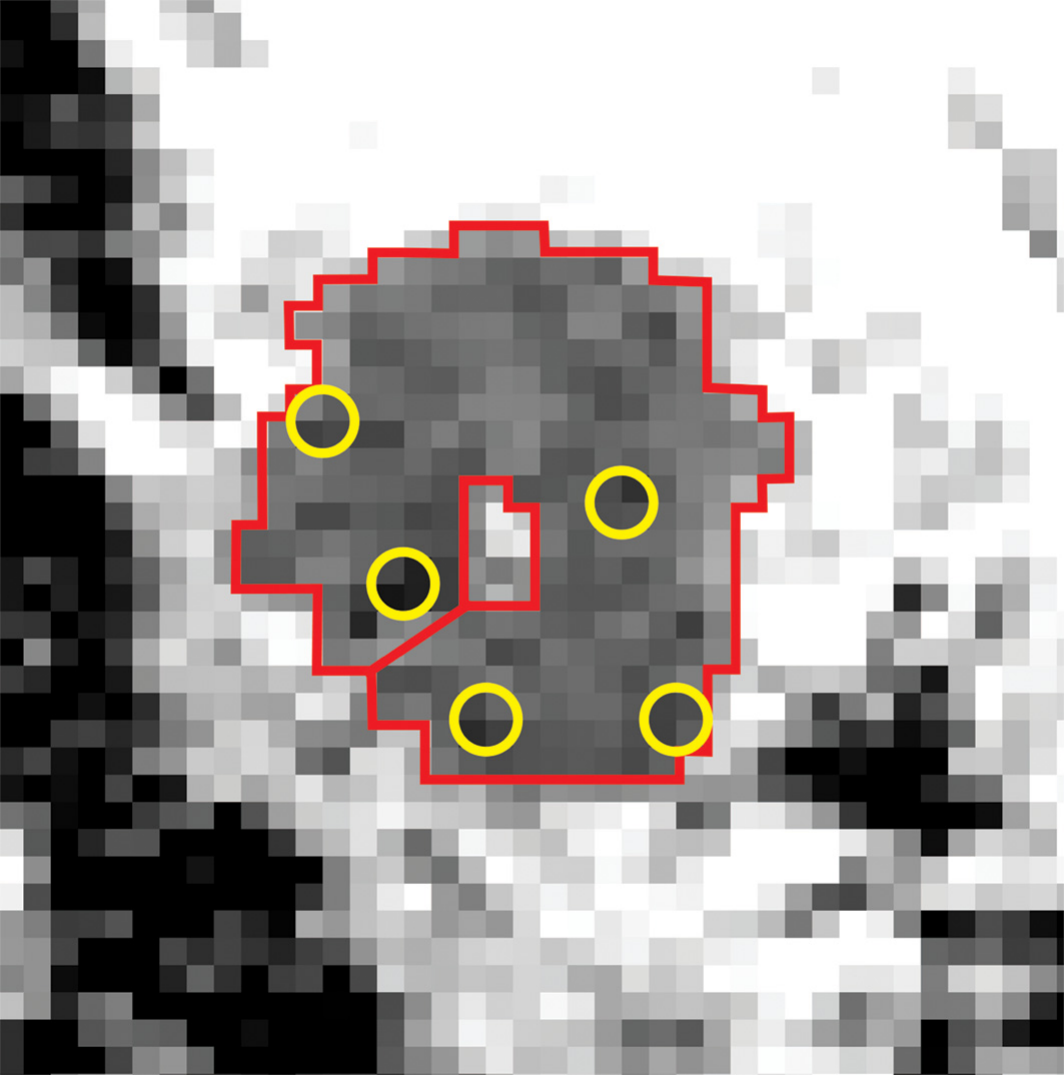
Whole lesion (*red*) and small (*yellow*) region of interest (ROI) placement on a diffusion-weighted image (DWI) of the right breast of a 41-year-old woman.

T1-weighted, T2-weighted, and DCE images and a crosshair tool (Sectra PACS, version 15.1.20.2, Sectra Workstation IDS7, Linköping, Sweden) were used to locate the lesion and to correctly position the ROI on ADC maps (Fig 1A and 1B). ROIs were placed on the hypo- or hyperintense lesions (N = 137) on ADC maps with a definitive demarcation from parenchyma and fat. A total of 137 lesions were included. First, one ROI was drawn polygonally to cover the entire lesion on the slice with the largest tumor diameter. Then, five smaller round ROIs (sized 3–4 pixels) were placed on the subregions with the lowest signal intensity inside the solid tumor on the ADC map on the same slice (Fig 2). Cystic, necrotic, fatty, and hemorrhagic areas were carefully avoided (Table 3). In addition, a large round ROI was drawn to include a portion of healthy fibroglandular tissue at the nipple level. Two observers (with 6 months and 2 years of experience in breast MRI analysis) independently evaluated all breast DWI data blinded to histopathological information using ImageJ software (version 1.47, Wayne Rasband, National Institutes of Health, Bethesda, MD, USA) [29]. Observer 1 evaluated primary lesions twice, with a 4-month interval between the measurements. Before the measurements were obtained, a senior consultant confirmed proper lesion localization for all lesions.

**Table 3:** Mean apparent diffusion coefficient (ADC) values (× 10^−3^ mm^2^s^-1^) of 3.0-Tesla using MRI from normal parenchyma, benign lesions, and malignant lesions, and ADC values’ utility in tumor characterization. A review of the literature.

	Bogner et al (2009)	Cakir et al (2013)	Park et al (2015)	Dong et al (2014)	Nogueira et al (2014)
Field strength and manufacturer	3T (Siemens)	3T (Philips)	3T (Siemens)	3T (GE)	3T (Siemens)
N	51	52	110	87	53
B values (mm^2^s^-1^)	500, 850	0, 50, 850, 1000, 1500; lower b range 0–1000 and higher b range 0–1500	0, 100	0, 800	50, 200, 400, 600, 800, 1000, 2000, 3000
MRI sequence order	DWI prior to DCE	DWI prior to DCE	UN	UN	DWI prior to DCE
ROI type and placement	Three-dimensional large ROI, avoiding fatty and necrotic tissue	Average of automatic double reading; having two combined b values; excluding hemorrhagic, cystic, and necrotic areas	Average of manual reading repeated 2–5 times; having two combined values; excluding hemorrhagic, cystic, and necrotic areas	Mean value of 3 round ROIs avoiding necrosis and hemorrhage, size 10 to 20 mm^2^	ROI placed on the area of highest hyperintensity on DCE, size 10 mm^2^
ADC breast parenchyma	1.87±0.22	Lower: 1.66±0.34, higher: 1.48±0.30	1.51±0.29	1.696	1.99±0.27
ADC benign lesion	1.51±0.22	Lower: 1.27±0.37, higher: 1.20±0.40	1.41±0.56	NS	1.71±0.35
ADC malignant tumor	0.99±0.18	Lower: 0.82±0.07 and b range 0–1500: 0.82±0.08	0.88±0.15	1.065	1.08±0.25
ADC cut-off for malignancy	1.25	b range 0–1000: 1.23, higher 1.12	NS	NS	1.41
Sensitivity (%)	96	Lower: 92.9, higher: 96.2	NS	NS	94.3
Specificity (%)	94	Lower: 54.5, higher: 59.1	NS	NS	87.5
Positive Predictive Value (%)	NS	Lower: 72.2, higher73.5	NS	NS	91.7
Negative Predictive Value (%)	NS	Lower: 85.7, higher: 92.9	NS	NS	91.3
Accuracy (%)	95	NS	NS	NS	91.5

T = tesla

ROI = region of interest

N = number of patients

NS = not studied

DWI = diffusion-weighted imaging sequence

DCE = dynamic contrast-enhanced imaging sequence

UN = unspecified.

### Histopathological Analysis

Before or after MRI, 14-G core needles were used to obtain histological samples from all lesions in which malignancy was suspected. Ultrasound-guided axillary lymph node (ALN) biopsy or sentinel lymph node biopsy (SLNB) were obtained according to local clinical practice [30]. If the ALN biopsy results revealed metastasis, the patient underwent ALN dissection.

Malignant lesions were further dichotomized into non-invasive premalignant and invasive tumors., Associations between ADC values and prognostic factors were analyzed in the invasive subgroup (N = 104). Carcinoma cells were considered negative for the estrogen receptor (ER) and the progesterone receptor (PR) if immunohistochemically determined expression was <10% and positive if expression was ≥10% [12]. HER2 gene amplification status was determined by clinically validated silver in situ hybridization. Immunohistochemically (Ki-67) determined proliferation was considered low (<10%), moderate (10–20%), or high (>20%). Lesions were regarded as triple negative if ER, PR, and HER2 statuses were negative. Invasiveness into lymph ducts, blood vessels or epidermis was recorded for carcinomas. Metastasis and/or micrometastasis to ALNs were reported by the pathologist. Low aggressiveness was defined as grade 1 and high aggressiveness as grade 2 or 3. The Nottingham Prognostic Index scores and categories [31] (NPIS) were calculated according to the pathologist’s report. Carcinomas were graded as stage Tis, 1, 2, 3, or 4 according to the guidelines of the National Cancer Institute [32].

### Statistical Analysis

The mean ADC values from the WL-ROI and the S-ROI that provided the lowest mean ADC value were selected. Continuous variables are presented as mean±standard deviation (SD), and categorical variables are presented as absolute values and percentages. Based on the Kolmogorov-Smirnov test, the Mann-Whitney U test for abnormally distributed nonparametric values were employed to compare ADC values between dichotomous groups and to evaluate the statistical difference between the ADC values in WL-ROIs and S-ROIs. The Kruskal-Wallis test was used to compare three or more groups. Spearman’s correlation coefficient was used to investigate the associations between continuous variables (ADC values, the Nottingham Prognostic Index score, size and age).

Receiver operating characteristic (ROC) curves were used to determine the optimal ADC thresholds for discriminating carcinomas from benign lesions. Sensitivities, specificities, positive and negative predictive values (PPVs and NPVs, respectively) and overall accuracies were calculated for both observers with the Bayes’ formula. Intra- and interobserver agreement were analyzed with the Kappa test and the intra-class correlation coefficient (ICC). McNemar's test was used to evaluate the differences in diagnostic performance between ROI types in mass and non-mass like enhancing (NMLE) breast lesions. Statistical significance was set at *P*≤0.05, and high statistical significance was set at *P*<0.01. Data was analyzed using SPSS for Windows (version 22, 1989–2013 SPSS Inc., Chicago, USA).

## Results

The study included 112 women (mean age 56.8±11.6 years, range 28–82 years) with a total of 137 biopsied DWI-visible lesions, of which 104 (75.9%) were malignant, 10 (7.3%) were premalignant (ductal carcinoma in situ), and 23 (16.8%) were benign (Table 1). One hundred eleven (81.0%) lesions were mass lesions and 26 (19.0%) were lesions with NMLE. Nine lesions with NMLE (34.6%) were invasive cancers. Mean tumor size was 2.33±1.64 cm (range 0.5–11.0 cm). Intraobserver reproducibility of the ADC measurements in primary lesions (N = 113) proved to be substantial for both WL-ROIs (κ = 0.683; ICC 0.817) and S-ROIs (κ = 0.732; ICC 0.707). Interobserver reproducibility (N = 137) was substantial (WL-ROIs: κ = 0.616 (P<0.001), ICC 0.831 (P<0.001); S-ROIs: κ = 0.784 (P<0.001); ICC 0.589 (P<0.001)). The mean ADC values were 1.02±0.30 × 10^−3^ mm^2^s^-1^ for parenchyma. Using the method proposed by Bogner et al. [17], the mean contrast-to-noise ratio (CNR) was -2.5±1.9 in a sample of 10 randomly selected lesions.

### Diagnostic Performance of ADC Values

**Fig 3:**
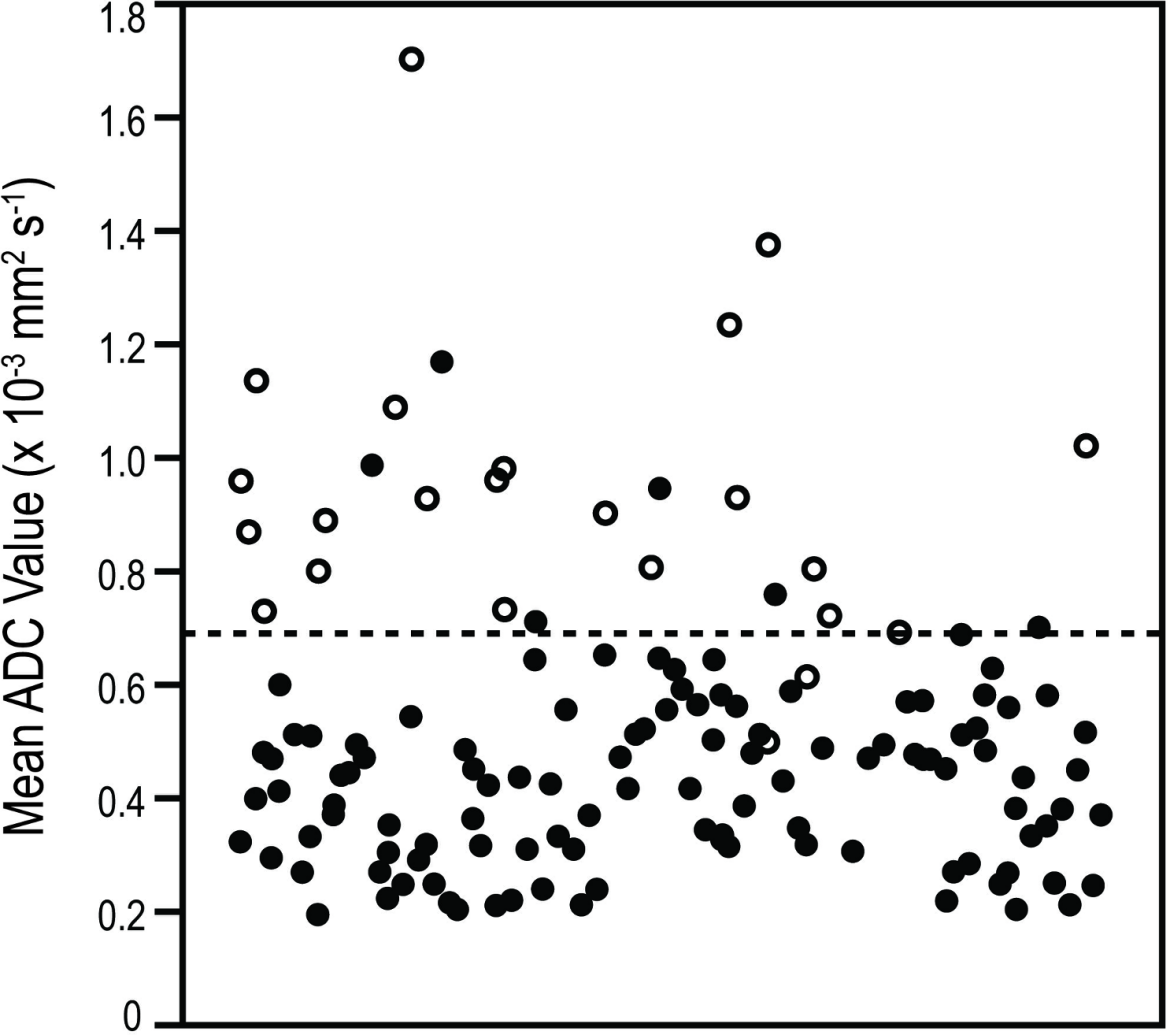
Distribution of apparent diffusion coefficient (ADC) values in benign and malignant lesions in a) whole lesion and b) small regions of interest (ROIs). ADC values (× 10^−3^ mm^2^s-^1^) of individual lesions are presented on the Y-axis. Black dots represent malignant lesions, and empty circles represent benign lesions. The line indicates the used cut-off values, 0.87 × 10^−3^ mm^2^s^-1^ for whole lesion ROIs and 0.69 × 10^−3^ mm^2^s^-1^ for small ROIs.

ADC values in malignant and benign lesions are presented in Table 2 and Fig 3. For both observers, both WL-ROIs and S-ROIs resulted in mean ADC values that were significantly lower in malignant lesions than in benign lesions (P<0.001, Table 2). Diffusion was more restricted in invasive than in premalignant lesions (P<0.001) and more restricted in premalignant lesions than in benign lesions (WL-ROIS: P = 0.013; S-ROIS: P = 0.022).

### Comparison of WL-ROIs with S-ROIs

**Fig 4:**
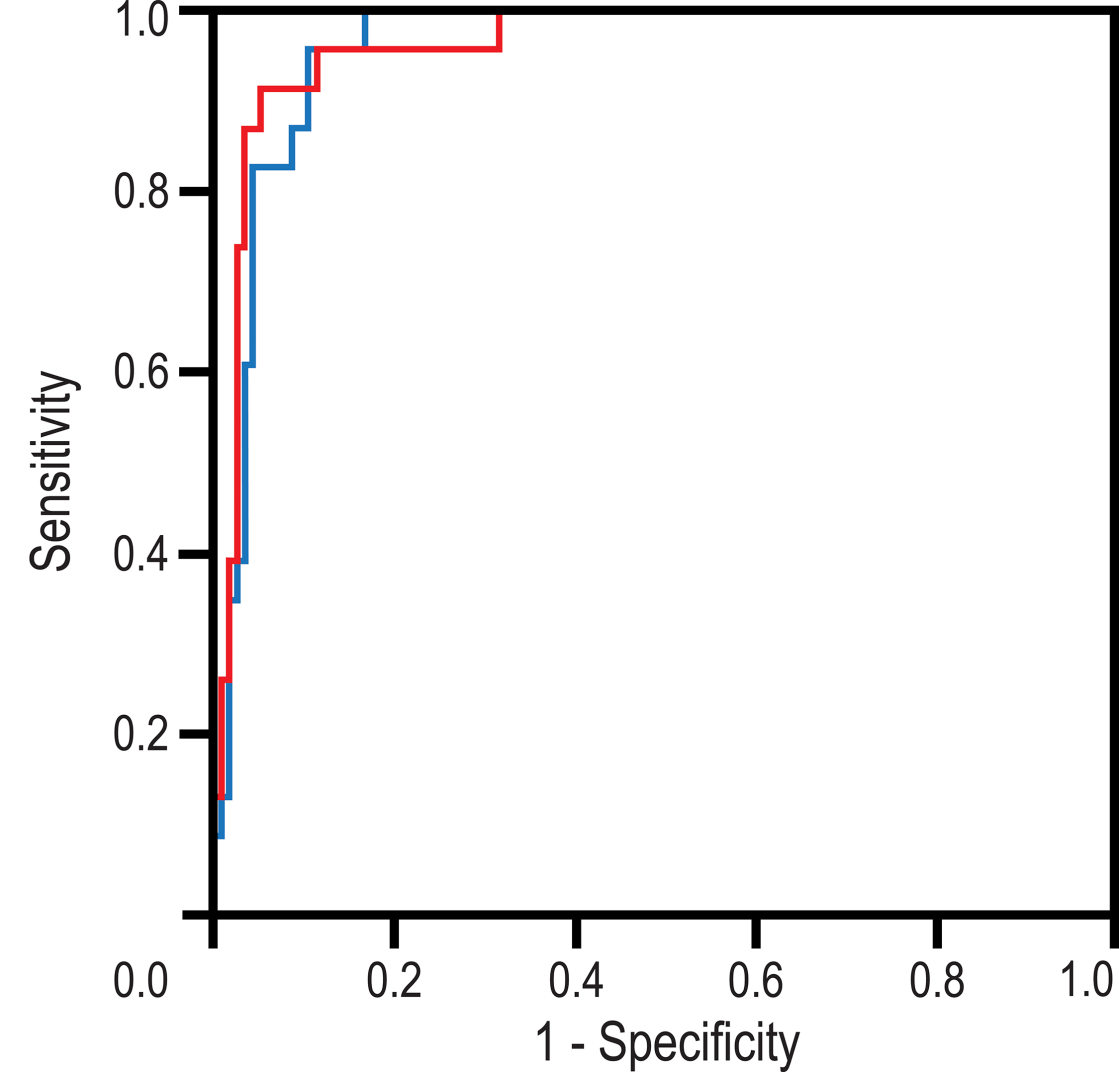
Receiver operator characteristic curves of whole lesion (*red line*) and small (*black line*) regions of interest (ROIs). Area under the curve values are 0.957 (whole lesion ROI) and 0.962 (small ROI).

Mean ADC values were significantly lower in the S-ROIs (0.52±0.26 × 10^−3^ mm^2^s^-1^) than in the corresponding WL-ROIs (0.69±0.27 × 10^−3^ mm^2^s^-1^, P<0.001). Using WL-ROI and applying a cut-off value of 0.87 × 10^−3^ mm^2^s^-1^ achieved 95.7%/94.4% sensitivity, 89.5%/47.6% specificity, 63.6%/88.5% PPV, 98.1%/66.7% NPV, and 89.8%/83.1% overall accuracy for observers 1 and 2, respectively. Using S-ROI and a cut-off value of 0.69 × 10^−3^ mm^2^s^-1^ achieved 91.3%/95.0% sensitivity, 94.7%/60.0% specificity, 77.8%/94.1% PPV, 98.2%/64.3% NPV, and 94.2%/92.6% overall accuracy. Area under curve values were 0.957/0.847 for WL-ROIs and 0.962/0.892 for S-ROIs (Fig 4). In mass lesions, the S-ROI provided superior differentiation of invasive carcinomas from benign lesions when compared with the WL-ROI (observer 1: P = 0.031; observer 2: P = 0.003). However, ROI type played no role in the characterization of breast lesions with NMLE (P = ns for both observers).

### Association between ADC Values and Prognostic Factors

When S-ROIs were used, ADC values were lower in high-grade tumors than in low-grade tumors, in carcinomas with metastasis to ALN vs. metastasis-free nodes and in carcinomas with lymphovascular invasion compared with tumors that lack this kind of invasiveness. In addition, these lower ADC values correlated inversely with the NPIS (P<0.05) but not with categorized Nottingham Prognostic Index. None of these differences were observed when WL-ROIs were employed. ADC values were lower in tumors with higher vs. lower stage and in PR-positive vs. PR-negative tumors in both ROI types (Table 2). No association was observed between ADC values and other receptor statuses, proliferation index, tumor size, or patients’ ages (Tables 2 and 4).

**Table 4:** Previous publications that address the correlation between apparent diffusion coefficient (ADC) values and prognostic factors in breast cancer.

	Razek et al 2010 (NMR in Biomedicine)	Tan et al 2014 (Clinical Radiology)	Nogueira et al 2014 (Clinical Radiology)	Kim et al 2009 (Journal of Magnetic Resonance Imaging)	Costantini et al 2010 (Clinical Radiology)	Choi et al 2012 (The British Journal of radiology)	Jeh et al 2011 (Journal of Magnetic Resonance)	Kamitani et al 2013 (Magnetic Resonance Med Sci)	Nakajo et al 2010 (Eur J Nucl Mol Imaging)	Present study
N patients (lesions)	57 (57)	50(44)	53 (59)	94 (67)	136 (162)	335 (335)	181 (107)	130 (81)	44 (44)	112 (137)
Magnetic resonance field strength and manufacturer	1.5-T (Siemens)	3.0-T (GE)	3.0-T (Siemens)	1.5-T (GE)	1.5-T	1.5-T (Siemens)	1.5-T (Siemens) and 3.0-T (Siemens)	1.5-T (Philips)	1.5-T (Philips)	3.0-T (Philips)
B values (mm2/s)	200, 400	500, 1000	50, 200, 400, 600, 800, 1000, 2000, 3000	0, 85	0, 1000	0, 1000	1.5-T: 0 and 1000, 3.0-T: 0 and 750	0, 500, 1000	0, 1000	0, 200, 400, 600, 800
MRI sequence order	UN	DWI prior to DCE	DWI prior to DCE	UN	DWI prior to DCE	UN	UN	UN	DWI prior to DCE	DWI prior to DCE
ROI type and placement	Manually placed, six small ROIs (minimum 3 pixels) on hypointensity; excluding hemorrhagic, cystic, parenchymal, and necrotic areas	Manually placed, smaller than tumor	Three randomly placed ROIs, size 0.5 cm, on hypointensity	ROI size 0,10 cm^2^, ROIs on hypointensity	ROI size 10±2mm, avoiding fatty and necrotic tissues	Whole tumor	Three measurements on hypodense areas; cystic and necrotic areas were avoided	Maximally sized; excluding necrotic and cystic areas	Large ROI; smaller than tumor; excluding cystic and necrotic areas	Five small ROIs (4 pixels) on hypodensities; lowest ADC value used and large ROI; excluding hemorrhagic, necrotic, fatty, and cystic areas
ADC values’ correlation (P) to:										
Age	NS	NS	NS	NC	NS	0.028	NC	NS	NS	NC
Size	P = 0.001	NC	NS	NC	NS	NC	NC	NC	NC	NC
Lymph node metastasis	P = 0.001	NC	NS	NS	NS	NC	0.964; NC	0.017	0.029	Correlation (small ROI) (P = 0.03)
Grade	0.001	NC	NS	NC	Inverse correlation between grades G1-G3 (0.001); Correlation with high and low aggressiveness. (0.001)	NC	NC	NS	0.001	Correlation with high and low aggressiveness (small ROI)(P = 0.006)
ER	NS	NC	NS	NC	NS	0.003	0.027	0.005	NC	NC
PR	NS	NC	NS	NC	NS	0.032	NC	0.048	NC	Correlation (small and whole lesion ROI)(P = 0.046 and P = 0.008, respectively)
Ki-67	NS	NC	NS	NC	NS	NC	NC	NS	NS	NC
HER2	NS	NC	NS	NC	NS	NC	0.018	NC	NC	NC
Triple negative malignancy	NS	NS	NS	NC	NS	NS	NS	NS	NS	NC

N = number

NS = not studied

DWI = diffusion-weighted imaging sequence

DCE = dynamic contrast-enhanced imaging sequence

UN = unspecified

NC = no correlation

P = P-value

ROI = region of interest

G = grade

ER = estrogen receptor

PR = progesterone receptor

Ki-67 = proliferation marker.

## Discussion

The results of this study show that 3.0-T DWI possesses considerable ability to differentiate between malignant and benign breast lesions. The ADC values also differentiated between invasive and in situ carcinomas. Use of both WL-ROI and S-ROI yielded good diagnostic performance and substantial reproducibility, although with significantly different cut-off values. S-ROI might be preferred over WL-ROI because it was more specific in the classification of malignant and benign lesions and was more frequently associated with prognostic factors.

According to previous literature, when employing 3.0-T MRI, the mean values for malignant and benign lesions vary considerably (0.792–1.455 × 10^−3^ mm^2^s^-1^ and 1.200–1.710 × 10^−3^ mm^2^s^-1^, respectively), which reflects the lack of standardization regarding ADC measurement methods (Table 3). Methods of ROI analysis should be standardized before cut-off values are selected for clinical practice. In the present study, the optimal cut-off values for malignancy obtained during ROC analysis (0.87 × 10^−3^ mm^2^s^-1^ for WL-ROIs and 0.69±0.27 × 10^−3^ mm^2^s^-1^ for S-ROIs) proved to be lower than the majority of those proposed in the literature (1.12–1.41 × 10^−3^ mm^2^s^-1^) (Table 3). Our protocol, in terms of contrast-to-noise ratio was comparable to the results presented by Bogner et al. [17]. Use of varying b values may partly explain the aforementioned differences [33]. No consensus exists on imaging technique and appropriate b-values. B-values of 50 and 850 s/mm^2^ on 3.0-T MRI were defined as optimal for breast tumours in a previous study evaluating the diagnostic quality of DWI [17]. Other explanation is that in the present study, the DWI sequence was consistently collected after the contrast medium administration. It has been previously suggested that DWI and DCE-MRI can be collected in any order without affecting the diagnostic criteria [34]. In a recent review by Dorrius et al, contrast medium had no significant effects on the ADC values (p≥0.08) [33]. However, Yuen at al suggested that postcontrast ADC values were lower than the precontrast values due to microperfusion effect [35]. It has also been proposed that the sequestration of gadolinium within the interstitial, extracellular space of the breast lesions would result in background gradients that reduce ADC values [36]. Janka et al concluded that DWI after the contrast medium administration could lead to improved lesion characterization [37].

We are not aware of studies that compare the effect of ROI type in breast lesion diagnostics in ADC analyses. Our results suggest that using S-ROIs placed on the most hypointense area of the ADC map instead of ROIs that cover the plausibly heterogeneous whole lesion can improve the specificity of lesion characterization, albeit at the expense of reduced sensitivity. Because high sensitivity (85–100%) can be achieved on DCE [4, 5], the improved specificity imparted by using S-ROIs may be of more clinical relevance and may reduce the number of unnecessary biopsies [6]. Our results suggest that in mass lesions, the presence of low ADC values even in small areas within the tumor may indicate further meticulous evaluation. Use of the most malignancy-suggestive kinetic curve in DCE is recommended by the BI-RADS^**®**^ guidelines [27]. Our proposed protocol parallels the DCE kinetic curve analysis, because both the ADC measurement and the worst appearing kinetic curve shape should be sampled from and reported for the S-ROI [27].

In mass lesions, choice of S-ROI instead of WL-ROI was more accurate and often indicative that further examinations were needed. However, the evaluation of lesions with NMLE using kinetic curves and ADC values is less reliable. In the present study, no statistical difference was observed between S-ROI and WL-ROI in lesions with NMLE. Because only a few of the lesions with NMLE were invasive cancers, results translate into differences in lesion cellularity and vascularization, and therefore are probably associated with different DWI and contrast enhancement behaviours. Furthermore, it has been speculated that these differences might be related to scarce cellularity or to contaminations of the background breast tissue [38].

ADC values are a three-dimensional representation of the mean diffusivity of the protons in water molecules. In breast lesions, ADC values are affected by tissue cellularity, fluid viscosity, membrane permeability, macromolecular structures, microvascularity and tumor blood flow [10, 11, 12, 39]. When characterizing a tumor, the use of a S-ROI represents the most aggressive tissue component analogous to the final histological diagnosis and minimizes the unintentional inclusion of fibroglandular tissue and fat [25].

The most important factors when assessing long-term survival are tumor size, axillary lymph node status, and histologic grade [40]. In clinical practice, ER, PR, HER2, and Ki-67 expression and status are applied. Promising correlations between ADC values and prognostic factors have been reported, although seldom when 3.0-T MRI is employed (Table 4).

In the present study, lower ADC values on S-ROIs correlated with ALN metastasis (P = 0.03) and higher tumor grade (P = 0.006); however, no such correlation was observed regarding WL-ROIs. There are no prior reports of any association between positive lymph node status, which is the most important single factor to predict long-term survival, and the primary tumor ADC values in 3.0-T MRI (Table 4). In contrast, Kamitani et al. in a report in which they used 1.5-T suggested that node positivity is seen in patients with high tumor ADC [14]. Direct proof of ALN metastasis using axillary ADC measurements remains challenging [41]. Lymphovascular invasion is another well distinguished factor known to associate with a lesion’s tendency to metastasize to axial lymph nodes. Notably, correlation with lymphovascular invasion was observed when S-ROI was used in carcinomas (P = 0.032), but not regarding WL-ROI. Our finding supports the results of Nakajo et al., which describe vascular invasion in tumors with lower ADC values, although they used a ROI that covered almost the whole lesion in 1.5-T MRI [15].

In agreement with our results, an inverse correlation between higher tumor grades and lower ADC values has been reported using 1.5-T MRI [11, 15]. However, contradictory results have also been described (Table 4). An association with tumor size was reported in a study of 57 invasive ductal carcinomas [7]. The present study uncovered no correlation with tumor size, in agreement with three other studies conducted using 3.0-T MRI (Table 4). Although the molecular predictive markers (intracellular receptors ER and PR, tumor proliferation marker Ki-67, and HER2) and their role in DWI have been studied, no consensus has been established (Table 4). Interestingly, PR expression correlated with lower ADC values in both S-ROIs and WL-ROIs. Lower ADC values have been correlated to ER positive [12, 14] and PR positive cancers [12, 13, 14]. In our patient sample, no association was observed between ADC values and ER or HER2 status or proliferation marker, a result concordant with most previous studies (Table 4).

We observed an inverse correlation between low ADC values and prognostic variables measured using both NPIS and TNM stage. The NPIS [31], which takes into account lesion size, ALN status, and grade, is used to predict 5-year breast cancer survival. TNM staging evaluates survival on the basis of tumor size, lymph node status, and found metastases [32].

One limitation of our study is the relatively small number of different lesion subtypes. Furthermore, the number of benign lesions is also rather small, and this group consists of widely varying lesions. The number of NMLE lesions is scarce, which results from the inclusion criteria; in situ carcinomas are not recommended for MRI evaluation according to the EUSOMA criteria. [28] Studies should be conducted with larger patient samples to verify the results, and a larger number of benign lesions are also needed. The present study aimed to standardize the use of a small subregion vs. whole lesion ROI for lesion characterization. In the future, other parameters to assess tumor characteristics such as ADC heterogeneity assessed by histogram and texture analyses and clustering methods are of interest. Also, the number of small ROIs to result in the best diagnostic ability and means to manage the measurements (i.e. by selecting the lowest value or averaged mean for statistical analyses) remain to be evaluated.

In conclusion, measurement of ADC values in 3.0-T MRI is a valuable tool to assess breast tumors and may help in tumor characterization. S-ROIs proved to be more specific than WL-ROIs, and were more frequently associated with the most important prognostic factors. Even small intratumoral pockets of reduced ADC values may indicate that further evaluation is needed, could provide a surrogate marker for tumor aggressiveness, and might be a helpful tool in tumor differentiation. Our results suggest the need for the standardization of ADC ROI measurement to be concordant with the DCE measurement. The ADC cut-off values differed significantly depending on measurement procedure, which should be recognized when results from the literature are adapted to clinical practice.
