# Correction: Diffusion-Weighted Imaging in 3.0 Tesla Breast MRI: Diagnostic Performance and Tumor Characterization Using Small Subregions vs. Whole Tumor Regions of Interest

**DOI:** 10.1371/journal.pone.0141833

**Published:** 2015-10-23

**Authors:** The PLOS ONE Staff

The first author’s name is spelled incorrectly. The correct name is: Otso Arponen. The correct citation is: Arponen O, Sudah M, Masarwah A, Taina M, Rautiainen S, Könönen M, et al. (2015) Diffusion-Weighted Imaging in 3.0 Tesla Breast MRI: Diagnostic Performance and Tumor Characterization Using Small Subregions vs. Whole Tumor Regions of Interest. PLoS ONE 10(10): e0138702. doi:10.1371/journal.pone.0138702
